# Comparative Assessment of the Activity of Racemic and Dextrorotatory Forms of Thioctic (Alpha-Lipoic) Acid in Low Back Pain: Preclinical Results and Clinical Evidences From an Open Randomized Trial

**DOI:** 10.3389/fphar.2021.607572

**Published:** 2021-02-24

**Authors:** Alessandra Pacini, Daniele Tomassoni, Elena Trallori, Laura Micheli, Francesco Amenta, Carla Ghelardini, Lorenzo Di Cesare Mannelli, Enea Traini

**Affiliations:** ^1^ Department of Experimental and Clinical Medicine, Anatomy and Histology Section, University of Florence, Florence, Italy; ^2^ School of Biosciences and Veterinary Medicine, University of Camerino, Camerino, Italy; ^3^ Department of Neuroscience, Psychology, Drug Research and Child Health (NEUROFARBA)-Pharmacology and Toxicology Section, University of Florence, Florence, Italy; ^4^ Section of Human Anatomy, School of Pharmacy, University of Camerino, Camerino, Italy

**Keywords:** neuropathic pain, thioctic acid, antioxidant, food supplement, neuroprotection

## Abstract

Peripheral neuropathies, characterized by altered nociceptive and muscular functions, are related to oxidative stress. Thioctic acid is a natural antioxidant existing as two optical isomers, but most clinically used as racemic mixture. The present study investigated the central nervous system’s changes which followed loose-ligation-derived compression of sciatic nerve, the putative neuroprotective role of thioctic acid and the pain-alleviating effect on low-back pain suffering patients. Loose ligation of the right sciatic nerve was performed in spontaneously hypertensive rats (SHR), a model of increased oxidative stress, and in normotensive Wistar-Kyoto rats (WKY). Animals with sciatic nerve ligation were left untreated or were treated intraperitoneally for 15 days with 250 μmol·kg^−1^·die^−1^ of (+/−)-thioctic acid; 125 μmol·kg^−1^·die^−1^ of (+/−)-thioctic acid; 125 μmol·kg^−1^·die^−1^ of (+)-thioctic acid lysine salt; 125 μmol·kg^−1^·die^−1^ of (−)-thioctic acid; 300 μmol·kg^−1^·die^−1^ pregabalin. Control SHR and WKY rats received the same amounts of vehicle. The clinical trial NESTIORADE (Sensory-Motor Neuropathies of the Sciatic Nerve: Comparative evaluation of the effect of racemic and dextro-rotatory forms of thioctic acid) examined 100 patients (49 males and 51 females aged 53 ± 11 years) dividing them into two equal-numbered groups, each treated daily for 60 days with 600 mg of (+/−)-thioctic acid or (+)-thioctic acid, respectively. The trial was registered prior to patient enrollment at EudraCT website (OSSC Number: 2011-000964-81). In the preclinical study, (+)-thioctic acid was more active than (+/−)- or (−)-enantiomers in relieving pain and protecting peripheral nerve as well as in reducing oxidative stress and astrogliosis in the spinal cord. Main findings of NESTIORADE clinical trial showed a greater influence on painful symptomatology, a quicker recovery and a better impact on quality of life of (+)-thioctic acid vs. (+/−)-thioctic acid. These data may have a pharmacological and pharmacoeconomical relevance and suggest that thioctic acid, above all (+)-enantiomer, could be considered for treatment of low-back pain involving neuropathy.

## Introduction

Neuropathic pain is a form of chronic pain caused by lesions to central or peripheral nervous system, which may be consequent to mechanical damage or diseases. It is characterized by altered nociceptive threshold and pain response, resulting in allodynia and hyperalgesia (Riego et al., 2018). The lumbosacral syndrome is a frequent neuropathic pathology described by a strong low back pain which may come from damage or irritation of sciatic nerve roots (Delitto et al., 2012). Treatment of its symptoms is still debated: the comparative evaluation of efficacy and tolerability of different drug categories (anti-inflammatory drugs, corticosteroids, antidepressants, anticonvulsants, muscle relaxants and opioids), concludes that most of the analyzed studies are poor quality and the data insufficient to provide guidance in the long-term treatment of the disease (Pinto et al., 2012; Schnitzer et al., 2004).

The excessive and unbalanced presence of reactive oxygen and nitrogen species causes oxidative stress, which alters the structure of the biomolecules and consequently induces neuronal damage (Adibhatla and Hatcher, 2010), inflammatory events and negative loop of excitotoxicity of afferent nociceptors, thus contributing to pain chronicization. Antioxidant agents, like thioctic (alpha-lipoic) acid, proved a therapeutic potential against neuropathy (Shay et al., 2009; Oyenihi et al., 2015).

Thioctic acid is a natural substance, synthetized *de novo* in mammalian mitochondria and existing as two optical isomers (+)-, endogenously produced and biologically active, and (−)-enantiomers. Racemic (+/−)-thioctic acid is sold worldwide as a registered drug or in nutraceutical market as dietary supplement and was reported to be a valid pharmacological agent in treating oxidative stress related diseases (Packer et al., 1995; Vasdev et al., 2000; Gomes and Negrato, 2014). Clinical studies showed that treatments with the racemic compound were able to reduce neuropathic low back pain (Memeo and Loiero, 2008; Ranieri et al., 2009). Even if the racemic mixture is the most widely used because of its stability, recent studies developed salt derivatives of (+)-thioctic acid with enough stability for a therapeutic use on its own (Ranieri et al., 2009; Amenta et al., 2010). Comparative studies revealed that (+)-thioctic acid displays a more pronounced activity than the racemic (+/−)-thioctic acid in several preclinical paradigms (Amenta et al., 2010; Lokhandwala, 2010). In the present study, we aimed to investigate if the (+)-thioctic acid is more active than its racemic congener on painful symptoms of sensory-motor neuropathies of the sciatic nerve in both a preclinical and clinical setting. The strain of spontaneously hypertensive rats (SHR), genetically harbouring hypertension and oxidative stress, was chosen and underwent to the loose ligation of the sciatic nerve (Tayebati et al., 2012). Pain relieving as well as neuroprotective and antioxidant effects of thioctic acid forms were compared to those of the reference drug pregabalin, a currently in-use anticonvulsant for treating chronic pain (Gilron et al., 2015; Xu et al., 2016). Based on the evidence from preclinical study, a clinical trial, named NESTIORADE (Sensory motor neuropathy of the sciatic nerve: Comparative assessment of the effectiveness of racemic and dextrorotatory forms of thioctic acid), was designed and conducted.

## Material and Methods

### Preclinical Study

#### Animals

Twenty-week-old male SHR (*n* = 42) and age-matched WKY (*n* = 42) rats were used. The animals were kept at 23 ± 1 °C with a 12 h light/dark cycle, light at 7 a.m. and fed with standard laboratory diet and tap water *ad libitum*. 24 h before the test, the animals were placed in the experimental room for acclimatization. All animal manipulations were carried out according to the Directive 2010/63/EU of the European Parliament and of the European Union council (September 22, 2010, amended by Regulation (EU) 2019/1010) on the protection of animals used for scientific purposes and to the ethical guidelines of the University of Florence, consistent with the Guide for the Care and Use of Laboratory Animals of the US National Institutes of Health (NIH Publication No. 85-23, revised 1996; University of Florence assurance number: A5278-01). Formal approval to conduct the experiments described was obtained from the Italian Ministry of Health and from the Animal Subjects Review Board of the University of Florence. Experiments involving animals were reported according to ARRIVE guidelines. All efforts were made to minimize animal suffering and to reduce the number of animals used.

#### Peripheral Mononeuropathy Rat Model

Neuropathy was induced in rats anaesthetized with 400 mg/kg chloral hydrate intraperitoneally (i.p.) Chronic Constriction Injury (CCI), according to the procedure described by (Bennett and Xie, 1988). Under aseptic conditions, the right common sciatic nerve was exposed at the level of the middle thigh by blunt dissection and four chromic cat gut ligatures (4-0, Ethicon, Norderstedt, Germany) were tied loosely around the nerve with about 1 mm spacing. After that hemostasis was confirmed, incision was closed in layers. After a period of recovery from surgery, animals were housed one per cage with free access to water and standard laboratory chow. Control animals were sham operated.

#### Animal Treatment

Thioctic acid, as (+/−)-compound, lysine salt (+)-enantiomer and (−)-enantiomer, was pursued from Sintactica (Milan, Italy). Compounds were solubilized in NaOH-supplemented physiologic solution and buffered to 7.4 pH by adding HCl. Different racemic and thioctic acid salt compounds were solubilized in saline and injected intraperitoneally (i.p.). Rats were treated for 15 days with intraperitoneal injection of 250 μmol·kg^−1^·die^−1^ (+/−)-thioctic acid (*n* = 6); 125 μmol·kg^−1^·die^−1^ (+/−)-thioctic acid (*n* = 6); 125 μmol·kg^−1^·die^−1^ (+)-thioctic acid lysine salt (*n* = 6); 125 μmol·kg^−1^·die^−1^ (−)-thioctic acid (*n* = 6); 300 μmol·kg^−1^·die^−1^ pregabalin (*n* = 6). Control CCI and SHAM (operated without ligating sciatic nerve) SHR and WKY rats (*n* = 6 each) received the same amounts of vehicle. To note, 250 μmol·kg^−1^·die^−1^ thioctic acid lysine salt (+)-enantiomer (about 90 mg/kg) can be converted in the human dosage of 569 mg (considering 70 kg body weight) accordingly to Reagan-Shaw et al. (2008) and Nair and Jacob (2016) using the equation [(rat dose mg kg^−1^/12.3) x 70].

#### Paw Pressure Test

One hour after the last drug administration, the nociceptive threshold was determined with an analgesimeter (Ugo Basile, Varese, Italy) (Leighton et al., 1988). A constantly increasing pressure was applied by a mechanical device to a small area of the dorsal surface of the paw, using a blunt conical probe. Mechanical pressure was increased until vocalization or a withdrawal reflex occurred while rats were lightly restrained. Vocalization or withdrawal reflex thresholds were expressed in grams. Rats scoring below 40 g or over 75 g during the test before drug administration (25%) were discarded. An arbitrary cut-off value of 250 g was adopted. The paw pressure test was repeated in a second session at 24 h after the first experiments.

#### Tissue Processing

One hour after completion of the paw pressure test, animals were sacrificed by cervical dislocation. The right sciatic nerve, was exposed, excised and the portion containing the ligature was removed. Contra-lateral nerves were also dissected out and a portion equivalent to that of ligated nerve was removed.

#### Paraffin Embedding and Staining

After animal sacrifice, sciatic nerves were fixed *in situ* with 4% formalin in phosphate buffered saline (pH 7.4). Following gradual dehydration in ethanol, nerve samples were embedded in paraffin (Diapath, Milan, Italy). Transverse 10 µm sections were cut on a microtome (Leica, RM 2145), and mounted on polylysine coated slides.

#### Sciatic Nerve Analysis: Histochemistry and Immunohistochemistry

Consecutive paraffin sections (10 µm thick) were stained alternatively with Mallory’s trichrome staining, to investigate morphology of different nerve components and occurrence of oedema and inflammatory infiltrates, or processed for immunohistochemistry techniques. Oedema and infiltrate were graded by an arbitrary scale starting from 1, mild infiltrate and oedema up to 10, severe infiltrate and widespread oedema. Sections were processed for 200 kDa neurofilament protein (NF) immunoreactivity, for Myelin Basic protein (MBP) or Glial fibrillary acidic protein (GFAP) immunohistochemistry using a mouse monoclonal antibody, as detailed in Table 1. Briefly, after deparaffinization and rehydration, sections were incubated in H_2_O_2_ 3% for 20 min, and in a blocking solution of bovine serum albumin (BSA) in phosphate buffer saline (PBS) 0.1 M pH 7.4 for 1 h at room temperature. Incubation with primary antibodies was performed over night at 4 °C at condition detailed in Table 1. After three washes in PBS, sections were incubated in a goat antimouse-biotinylated secondary antibody. The product of the immune reaction was revealed using a biotin–streptavidin immunostaining kit (Vectastain ABC Kit Elite, Vector, Cat. No. PK 6100) and 3.3′-diamino benzidine (DAB) as a chromogen (DAB peroxidase substrate, Vector Cat. No 4100). After washing, sections were then dehydrated in ethanol, mounted in mounting medium and observed under a light microscope. Control sections were processed in the same way but using a non-immune mouse IgG instead of the primary antibody. These sections did not develop specific immunostaining (data not shown). Sections processed for immunohistochemistry were viewed under a light microscope connected to the screen of IAS 2000 image analyzer. The intensity of axonal NF immunostaining and the intensity of MBP immunostaining developed in myelin sheaths were assessed microdensitometrically with an image analysis system, calibrated to take as “zero” the background developed in sections incubated with a non-immune serum and “100” as the conventional value of maximum intensity of staining.

**TABLE 1:** Primary antibodies used in immunohistochemistry.

Primary antibody	Company Cat. No.	Dilution IHC
Anti-neurofilament 200kDa, clone RT-97 (NF)	Monoclonal antibody, Merck-Millipore (Cat. No. MAB5262)	1:500
Anti-myelin basic protein (MBP)	Monoclonal antibody, Merck-Millipore (Cat. No. NE1019)	1:500
Anti-glial fibrillary acidic protein (GFAP)	Monoclonal antibody, Merck-Millipore USA (Cat. No. MAB3402)	1:500
Anti-8-OHdG	Monoclonal antibody clone 2E2, Trevigen (Cat. No. 4354-MC-050)	1:250
Anti-mouse biotinylated	Polyclonal antibody, Merck-Millipore (Cat. No. AP124B)	1:200
Anti-mouse	Alexa fluor 488®, CellSignaling technology (Cat. No. #4408S)	1:100

#### Spinal Cord Analysis: Malondialdehyde (MDA) Levels and DNA Oxidation Status

In portions of spinal cord, oxidative stress indicators were evaluated: malondialdehyde (MDA) levels via thiobarbituric acid reactive substances (TBARS) kit (Cayman, Chemical Company, Ann Arbor, MI, United States Cat. No. 10009055); DNA oxidation by 8-OHdG immunohistochemistry. Paraffin sections of spinal cord were processed for 8-OHdG immunohistochemistry, using monoclonal antibodies as detailed Table 1. After deparaffinization and rehydratation, sections were incubated in a blocking solution of bovine serum albumin (BSA) in phosphate buffer saline (PBS) 0.1 M pH 7.4 for 1 h at room temperature. Incubation with primary antibodywas performed over night at 4°Cat condition detailed in Table 1. After three washes in PBS, sections were incubated in a biotinylated secondary antibody solution (Table 1).

#### Protein Oxidation Status: Western Blot Analysis of Carbonylated Proteins

Samples of spinal cord, taken from six rats for each group, were homogenized in lysis buffer, as previously described (Tayebati et al., 2017). We assessed protein carbonylation by treating equal amounts of protein according to protocol of OxyBlot Protein detection kit (Millipore, USA, Cat. No. S7150). The kit provides a system to perform the immunoblot detection of carbonyl groups introduced into proteins by oxidative reactions. As a consequence, carbonyl groups are introduced into the side chains of all proteins independently of the molecular weight. The samples were separated by 8% SDS polyacrylamide gel, transferred onto nitrocellulose and blotted with the specific antibodies of the kit that recognize all the oxidized protein with different molecular weight. Band intensities were measured by densitometry with IAS 2000 image analyzer (Biosystem, Rome, Italy).

### Clinical Study

NESTIORADE is a comparative open trial, approved by the Ethic Committee of “Azienda Ospedaliera Universitaria Maggiore della Carità, Novara” and “Aziende Sanitarie Locali” of Novara, Biella, Vercelli and Verbano Cusio Ossola (NEST 2009) and written informed consent was obtained from all subjects participating in the trial. The trial was registered prior to patient enrollment at https://eudract.ema.europa.eu (OSSC Number: 2011-000964-81; principal investigator: Prof. Francesco Pipino; date of registration: February 28, 2011). The study recruited 100 patients (49 males and 51 females, with a mean age of 53 ± 11 years) who met criteria for inclusion and agreed to participate at the trial. The number of participants were chosen to ensure a minimum statistical power of 90% and alpha of 5%, considering variance and effect of primary outcomes. Patients were divided by block randomization using a random generation number into two different groups of 50 subjects each and were assigned to a 600 mg/day treatment with (+/−)-thioctic acid (Group 1), or to a 600 mg/day treatment with (+)-thioctic acid (Group 2). Treatments lasted 60 days. Study was not controlled by placebo due to a request of the ethic committee, that did not allow to keep patients without therapy during pain condition. (+/−)-thioctic acid therapy was considered safe, effective and a good reference thanks to publications that highlighted the efficacy and safety of its use in similar pathologies (Memeo and Loiero, 2008; Ranieri et al., 2009).

Inclusion criteria were:

- Radiculopathy of the lower limbs,
- Diagnosis confirmed by CT or MRI,
- Unilateral or bilateral presentation,
- First event,
- Onset of symptomatology not exceeding 40 days.

Exclusion criteria were:

- Cognitive deficits or psychiatric disorders,
- Specific indication for surgical treatment of symptomatology,
- Poor compliance toward inclusion in the study,
- Concomitant neoplastic pathology,
- Chemotherapy or immunosuppressive treatment ongoing,
- Under treatment with thioridazine hydrochloride.

The evaluation of time loss/disappearance of symptoms was performed by using multidimensional scales designed for neuropathic pain (Xiong et al., 2015) listed below:

1. Neuropathy Symptoms and Change (NSC) (Dyck et al., 2002);
2. Neuropathy Impairment Score (NIS) (Dyck et al., 2002);
3. Neuropathic Total Symptom Score-6 (NTSS-6) (Bastyr et al., 2005).

To better assess the impact of the compared treatments on life quality of patients, the following parameters were evaluated:

1. Consumption of analgesics during the whole treatment period;
2. Quality of sleep.

**FIGURE 1:**
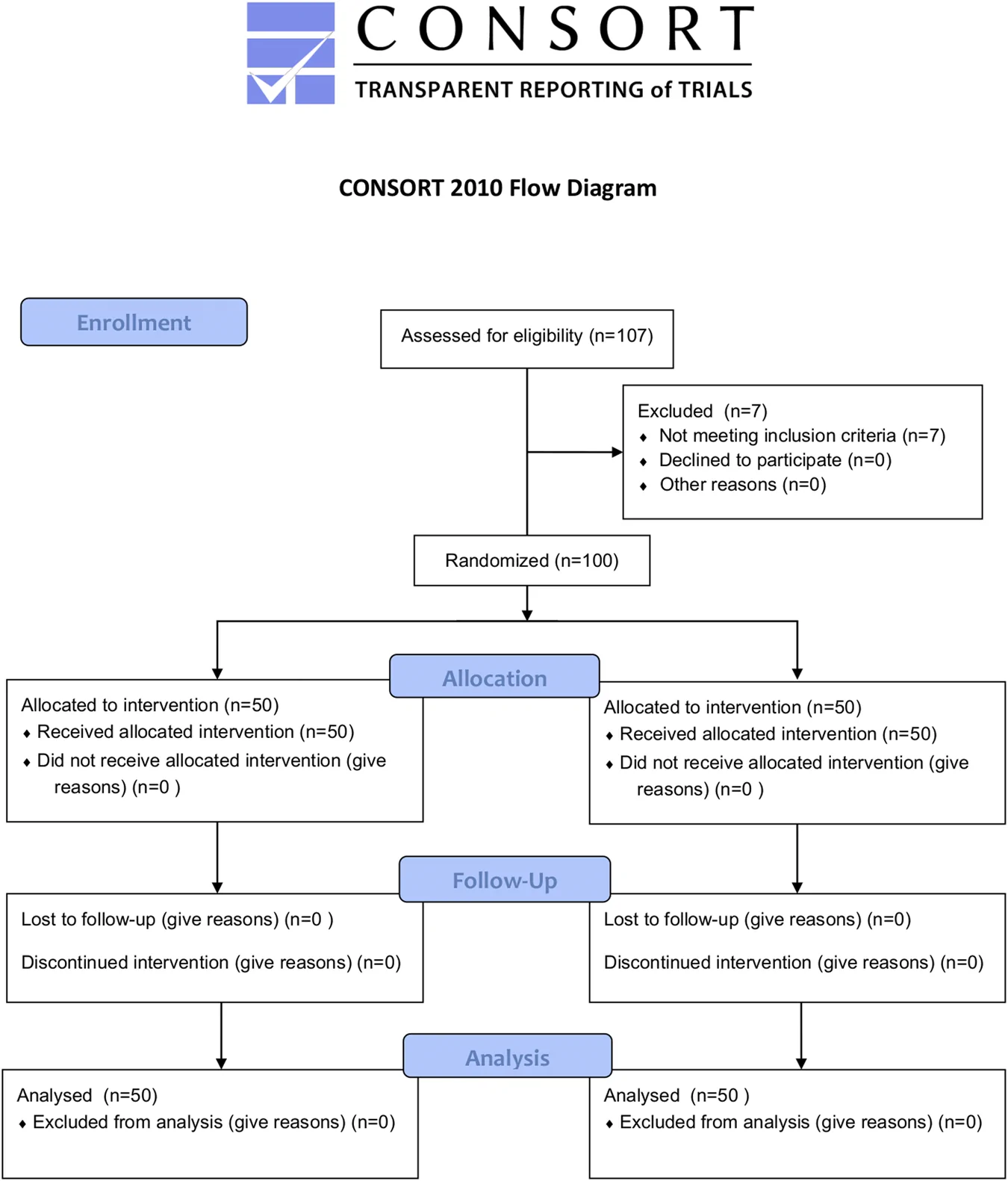
CONSORT flow diagram of the clinical study NESTIORADE (Sensory motor neuropathy of the sciatic nerve: Comparative assessment of the effectiveness of racemic and dextrorotatory forms of thioctic acid).

This manuscript adheres to the applicable CONSORT guidelines. (Figure 1).

### Statistical Analysis

All data of different parameters were expressed as mean ± S.D. (*n* = 6), calculated from single animal data. The significance of differences between groups of treatment was analyzed by analysis of variance (ANOVA) followed by the Newman-Keuls test, while for the analysis of the differences within the group over time, Student’ t-test for paired data were performed. For analyzing the different treatment over time a two way ANOVA for repeated measure was used. X-squared (X^2^) test was performed to evaluate differences in qualitative data. Data were collected by researchers blind to the treatments.

## Results

### Preclinical Results

#### Paw Pressure Test

**FIGURE 2:**
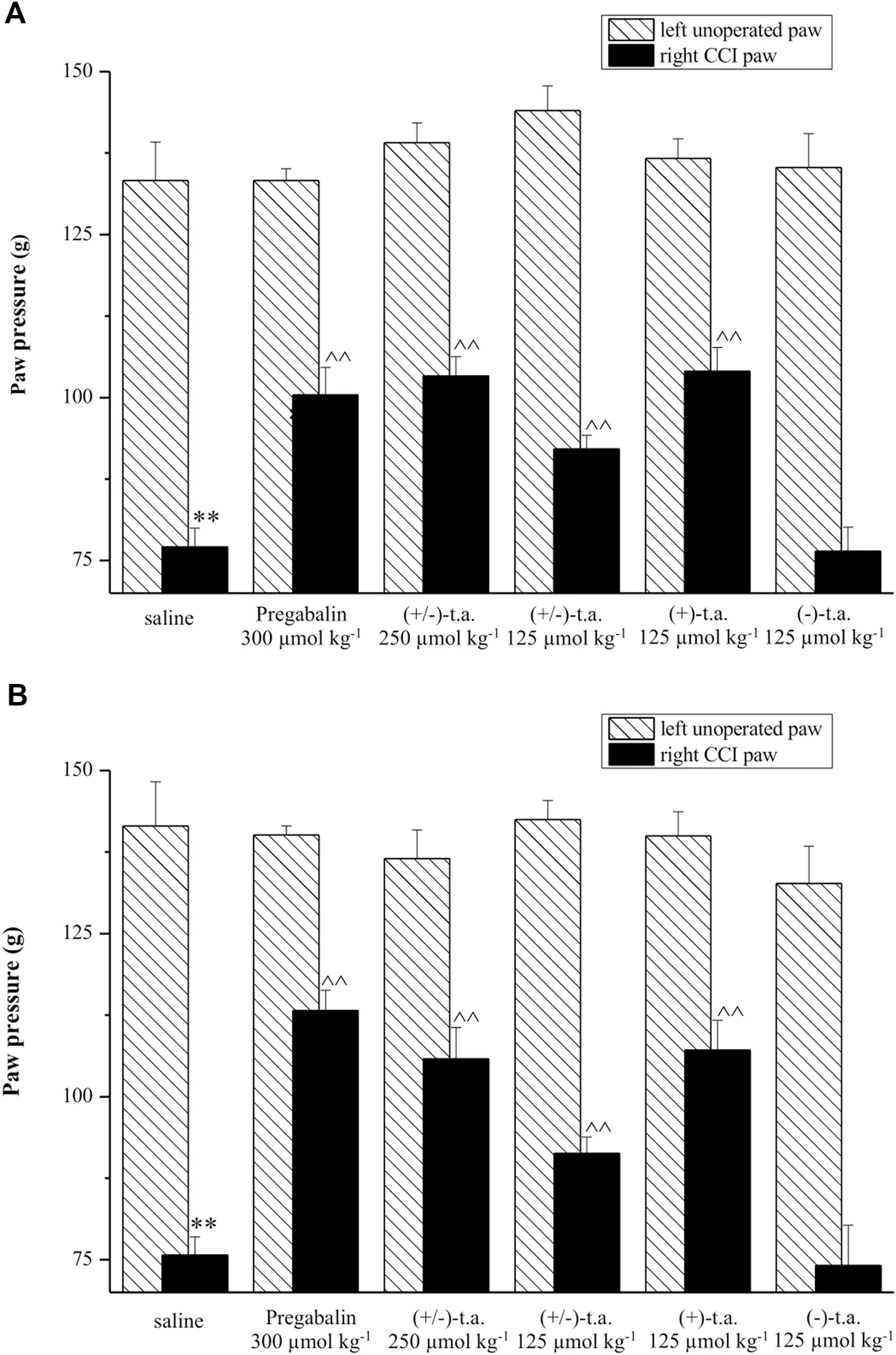
Paw pressure test. Paw pressure test on operated (CCI) and unoperated paws of WKY **(A)** and SHR **(B)** following treatment with 300 μmol·kg^−1^·die^−1^pregabalin, 250 and 125 μmol·kg^−1^·die^−1^ (+/−)-thioctic acid (t.a.), 125 μmol·kg^−1^·die^−1^ (+)- and (−)-thioctic acid or with vehicle (saline). Each experimental group is *n* = 6; data are expressed as mean ± SEM, in grams. **p < 0.01 vs the left unoperated paw value, ^ ^*p* < 0.01 vs CCI + saline solution.

CCI SHR and CCI WKY rats were treated intraperitoneally (i.p.) for 15 days with (+/−)-thioctic acid (125–250 μmol·kg^−1^·die^−1^) (+)-thioctic acid (125 μmol·kg^−1^·die^−1^), (−)-thioctic acid (125 μmol·kg^−1^·die^−1^) or pregabalin (300 μmol·kg^−1^·die^−1^), starting on the day of the surgery. Likewise, control SHAM and CCI animals were treated i. p. with vehicle (data not shown). At the end of the treatment, mechanical hypersensitivity was evaluated in all the experimental groups of both strains, WKY (Figure 2A) and SHR (Figure 2B), via paw pressure test on ipsilateral and contralateral paw (1 h after the last treatment). Vehicle-treated CCI animals presented an altered response to the noxious stimulus on the ipsilateral paw, tolerating a bit more than half of control weight; there was no significant difference in pain response between the strains in all the experimental conditions. No difference were reported between the left paw of vehicle-treated CCI and SHAM group. A beneficial effect was observed with repeated (+/−)-thioctic acid 250 μmol·kg^−1^·die^−1^ treatment (*p* = 0.0005), since it increased pain threshold of the ipsilateral paw of CCI animals; treatment with the half dose of (+)-thioctic acid produced the same positive effects (*p* = 0.0004). Half dose of racemic mixture induced a lower but however significant (*p* = 0.0008) improvement in pain sensitization. The analgesic activity of both compounds was comparable to the outcomes of pregabalin administrations; conversely, repeated treatment with (−)-enantiomeric isoform of thioctic acid was not active. Similar results were obtained repeating measurements 24 h after the last administration of thioctic acid forms (data not shown).

#### Morphological Analysis: Sciatic Nerve

**FIGURE 3:**
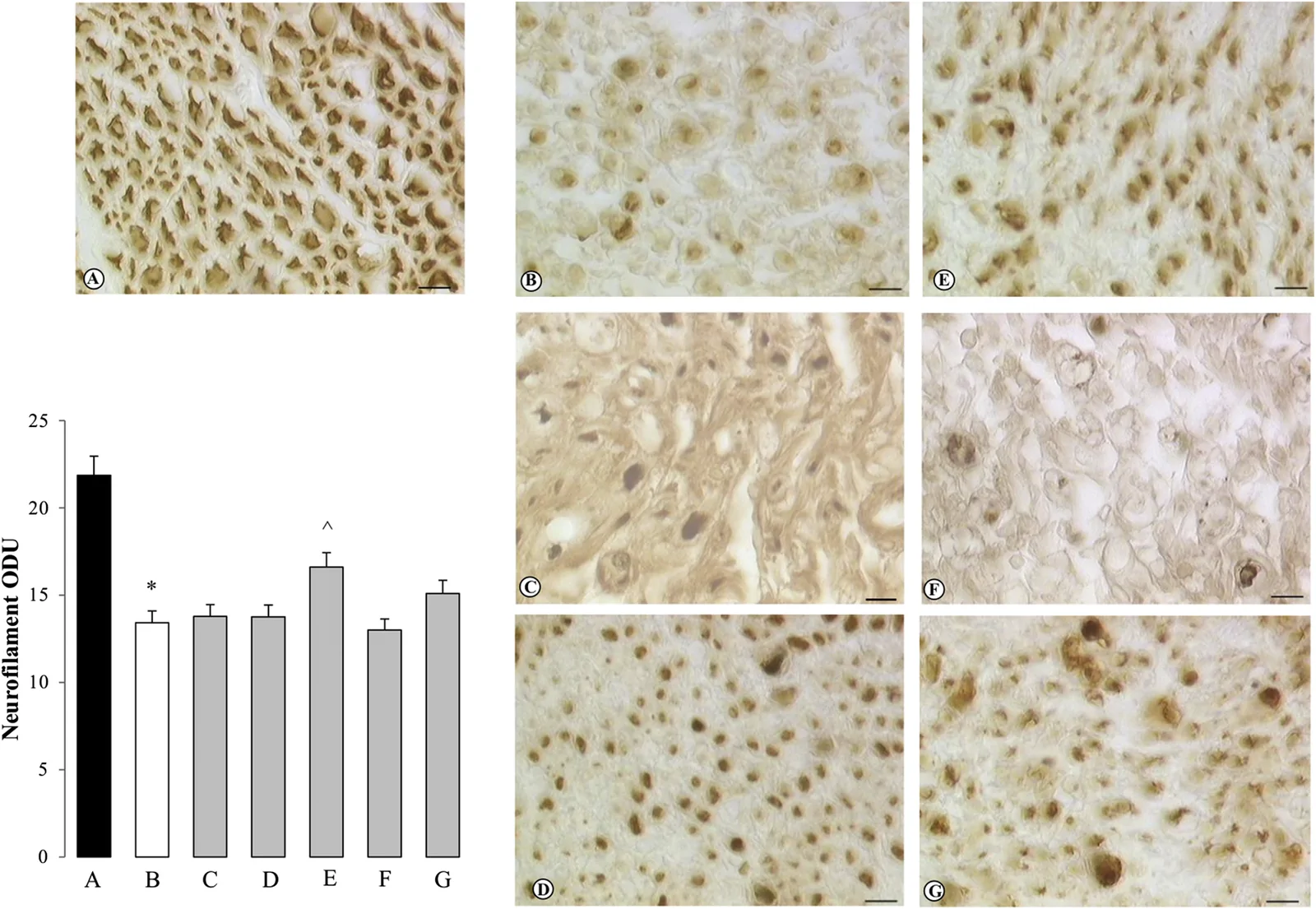
Sections of sciatic nerve processed for neurofilament immunohistochemistry. Treatments are indicated as follows: **(A)** SHAM SHR + saline solution; **(B)** CCI SHR + saline solution; **(C)** CCI SHR + (+/−)-thioctic acid 125 μmol·kg^−1^·die^−1^; **(D)** CCI SHR + (+/−)- thioctic acid 250 μmol·kg^−1^·die^−1^; **(E)** CCI SHR + (+)- thioctic acid 125 μmol·kg^−1^·die^−1^; **(F)** CCI SHR + (−)-thioctic acid 125 μmol·kg^−1^·die^−1^; **(G)** CCI SHR + pregabalin 300 μmol·kg^−1^·die^−1^. Left-bottom graph represents a densitometric analysis of the expression of neurofilament. Each experimental group is *n* = 6; data, expressed as Optical Density Unit (ODU), are the mean ± SEM. Calibration bar: 10 μm *p < 0.05 vs SHAM SHR, ^*p* < 0.05 vs CCI SHR + saline solution.

**FIGURE 4:**
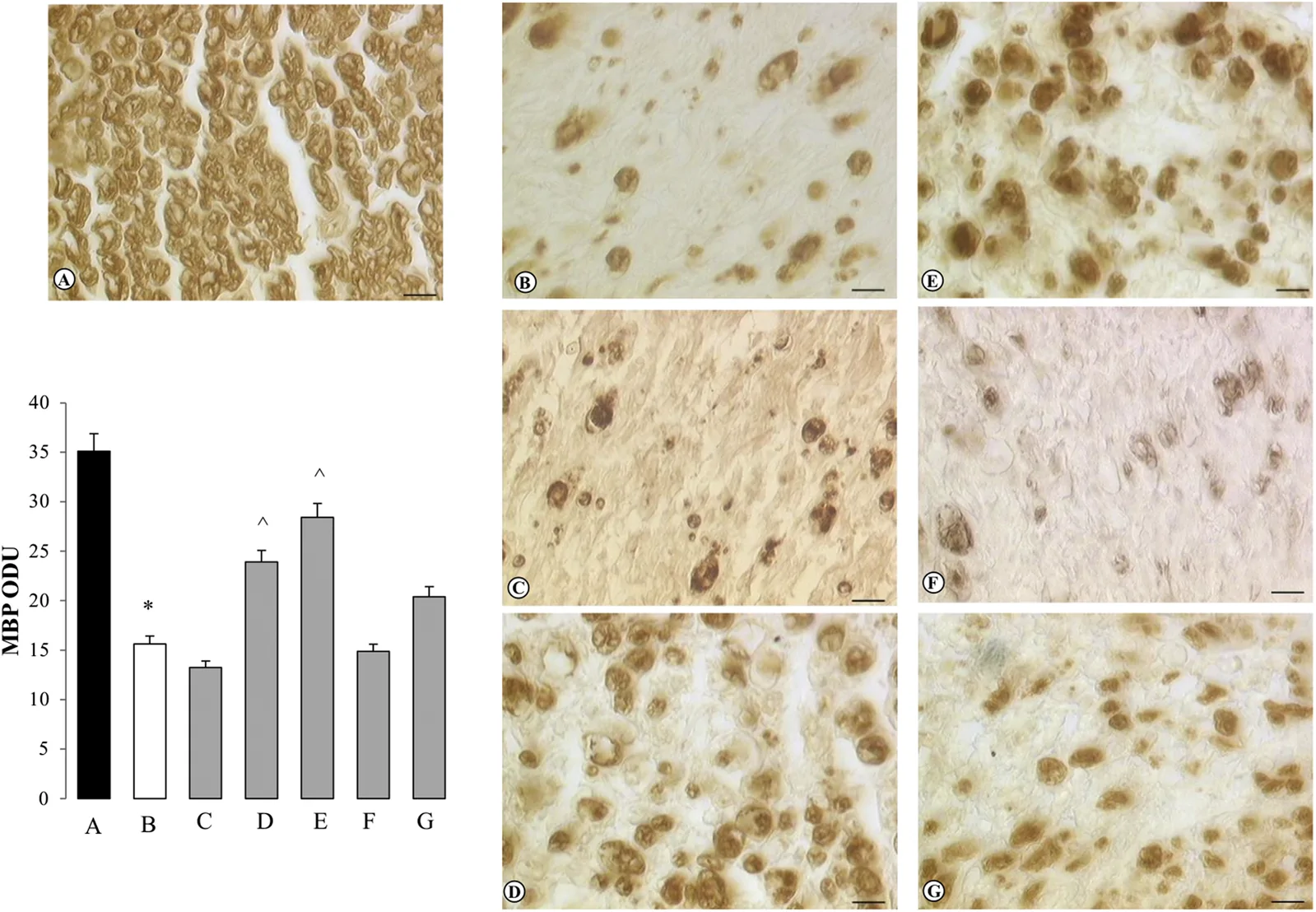
Sections of sciatic nerve processed for myelin basic protein (MBP) immunohistochemistry. Treatments are indicated as follows: **(A)** SHAM SHR + saline solution; **(B)** CCI SHR + saline solution; **(C)** CCI SHR + (+/−)-thioctic acid 125 μmol·kg^−1^·die^−1^; **(D)** CCI SHR +(+/−)-thioctic acid 250 μmol·kg^−1^·die^−1^; **(E)** CCI SHR + (+)-thioctic acid 125 μmol·kg^−1^·die^−1^; **(F)** CCI SHR + (−)-thioctic acid 125 μmol·kg^−1^·die^−1^; **(G)** CCI SHR + pregabalin 300 μmol·kg^−1^·die^−1^. Left-bottom graph represents a densitometric analysis of the expression of myelin basic protein. Each experimental group is *n* = 6; data, expressed as Optical Density Unit (ODU), are the mean ± SEM. Calibration bar: 10 μm *p < 0.05 vs SHAM SHR, ^*p* < 0.05 vs CCI SHR + saline solution.

After the behavioral tests, animals were sacrificed, both sciatic nerves and spinal cord tissue were explanted. Sciatic nerve sample tissues were stained and processed: Mallory’s trichrome staining (Supplementary Figure S1); immunohistochemistry for neurofilament (NF, Figure 3) and myelin basic protein (MBP, Figure 4).

Mallory’s trichrome staining on CCI right sciatic nerve highlighted a massive degeneration of myelinated and non-myelinated axons distal to the ligation site both in WKY (data not shown) and SHR rats (Supplementary Figure S1), induced by constriction injury. In CCI SHR sciatic nerve, Mallory’s trichrome staining of distal-to-ligation right sciatic nerve showed: a typical Wallerian degeneration with less compact oedematous axons and accumulation of inflammatory cells, absence or damage of myelin sheaths and a scarcely identifiable myelin-axon border (Supplementary Figure S1A). These changes were partly inhibited only by the treatment with (+/−)-thioctic acid 250 μmol·kg^−1^·die^−1^ (Supplementary Figure S1C) and (+)-thioctic acid (Supplementary Figure S1D). Administrations of (+/−)-thioctic acid 125 μmol·kg^−1^·die^−1^ (Supplementary Figure S1B) and (−)-thioctic acid (Supplementary Figure S1E) left the morphology of damaged nerve unaltered. Pregabalin injections (Supplementary Figure S1F), restored the morphology of the nerve countering the reduction of axon and myelin thickness in nerve fibers of the lesioned nerve.

Sections of sciatic nerve explanted from SHAM SHR were processed for NF immunohistochemistry: they developed a dark brown axonal staining, with a particularly intense immunoreaction in the external part of axons (Figure 3A). Reduced NF immunoreactivity was observed in the distal to ligation sciatic nerve from control (untreated) CCI SHR rats (Figure 3B) as also demonstrated by quantitative analysis, expressed as Optical Density Unity (ODU) in the left-bottom graph, that showed a significantly (*p* = 0.0006) decrease of quantitative immunoreaction (Figure 3, graph column B). Only the treatment with (+)-thioctic acid augmented axonal NF immunoreactivity in the distal part of the sciatic nerve in a significant way (*p* = 0.0025) (Figure 3, panel and graph column E). MBP immunostaining showed a physiological pattern of myelin organization in the SHAM operated rats, with dark brown immunoreactivity in the myelin sheaths (Figure 4A). As showed by quantitative analysis (Figure 4, left-bottom graph), a remarkable reduction (*p* = 0.0005) of MBP immunoreactivity was evident in the distal part of the ligated nerve (panel and graph column B). Treatment with racemic thioctic acid 250 μmol·kg^−1^·die^−1^ (panel and graph column D) and (+)-thioctic acid (panel and graph column E) significantly raised MBP immunoreactivity (*p* = 0.0003) in the distal part of ligated nerve, while the other compounds produced no improvements.

#### Morphological and Biochemical Analysis: Spinal Cord

**FIGURE 5:**
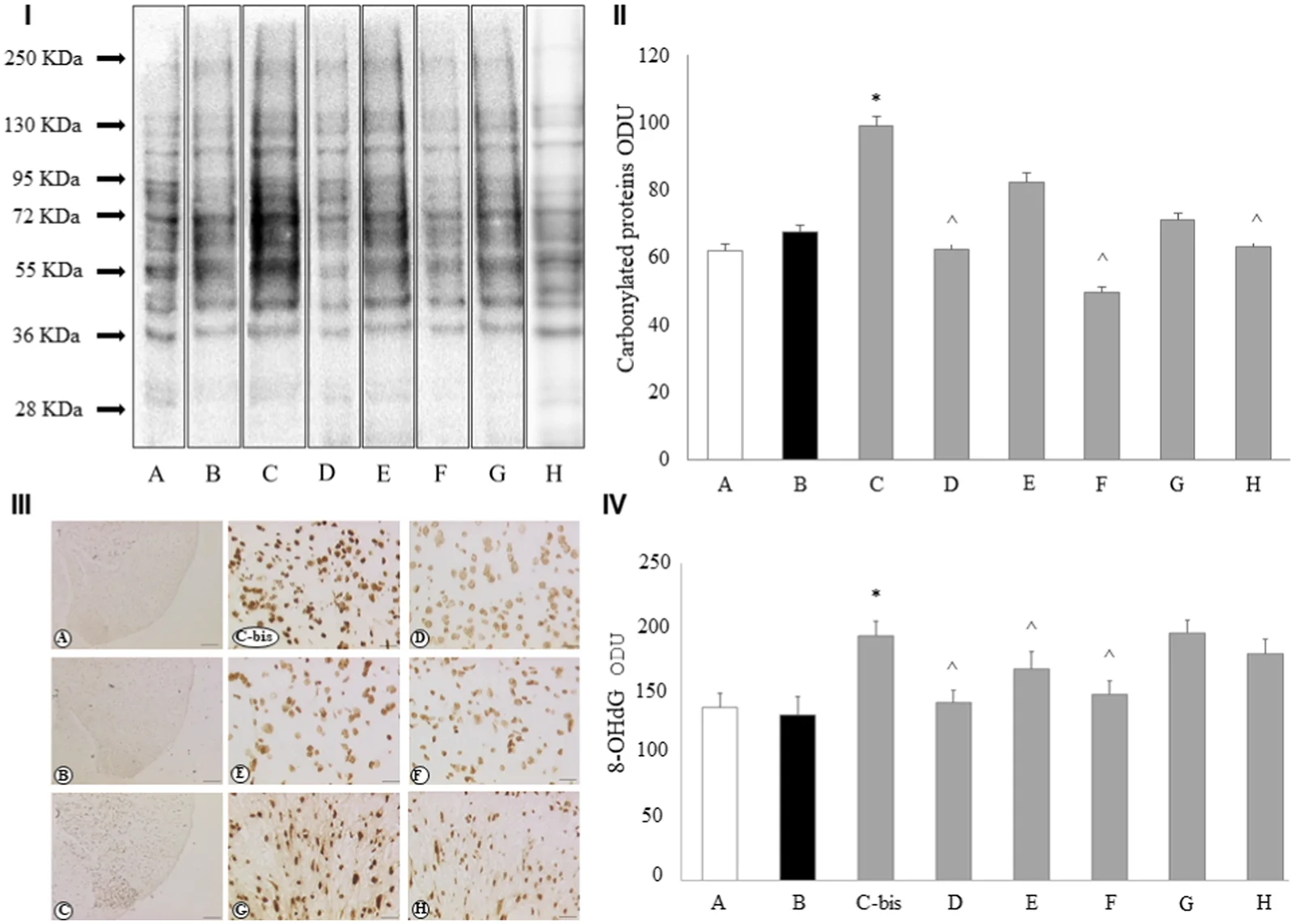
Oxidative stress status in the spinal cord (lumbar region L5). Carbonylated proteins blotting of the spinal cord **(I)** and its densitometric analysis **(II)**. Sections of dorsal horns of spinal cord (lumbar region L5) processed for immunohistochemistry of 8-OHdG **(III)** and densitometric analysis **(IV)**. Calibration bar of **(III)**: A-C 200 μm; Cbis-H: 25 μm; densitometric analysis was performed on 25 μm calibration bar-images. In all panels, treatments are indicated as follows: (A) SHAM WKY + saline solution; (B) SHAM SHR + saline solution; (C) Cbis) CCI SHR + saline solution; (D) CCI SHR + (+/−)-thioctic acid 250 μmol⋅kg^−1^⋅die^−1^; (E) CCI SHR + (+/−)-thioctic acid 125 μmol⋅kg^−1^⋅die^−1^; (F) CCI SHR + (+)-thioctic acid 125 μmol⋅kg^−1^⋅die^−1^; (G) CCI SHR + (−)-thioctic acid 125 μmol⋅kg^−1^⋅die^−1^; (H) CCI SHR + Pregabalin 300 μmol⋅kg^−1^⋅die^−1^. Data are expressed as mean ± SEM. *p < 0.05 vs. SHAM SHR + saline solution; ^ p < 0.05 vs. CCI SHR + saline solution.

In the spinal cord we investigated oxidative stress signals: the levels of malondialdehyde (MDA) (Supplementary Figure S2), protein carbonylation and 8-hydroxy-2′-deoxyguanosine (8-OHdG) (Figure 5: **I-II** and **III-IV**, respectively).

SHAM non-hypertensive and hypertensive rats had similar MDA levels, while a statistically significant raise (*p* = 0.034) was observed in the neuropathic SHR group (Supplementary Figure S2, column C) in comparison to control SHAM SHR (Supplementary Supplementary Figure S2, column B); all treatments were unsuccessful at recovery.

Densitometry analysis (Figure 5II) on blotted carbonylated protein (Figure 5I), showed that SHAM-operated WKY and SHR (A, B bands/columns) displayed the same low level protein oxidation, while CCI-induced neuropathy resulted in higher carbonylated protein values in spinal tissue of CCI SHR animals (C band/column). (+/−)-thioctic acid 250 μmol·kg^−1^·die^−1^ lowered carbonylated proteins to control values (D band/column), the same result was gained by dextrorotatory enantiomer administrations (F band/column) and both treatments resembled pregabalin effects (H band/column). Conversely, the treatment with (+/−)-thioctic acid 125 μmol·kg^−1^·die^−1^and with (−)-thioctic acid (E, G bands/columns) lead to a very slight non-significant reduction of protein oxidation. Expression of 8-OHdG in dorsal horns of spinal cord was investigated through immunohistochemistry and related densitometric analisys (Figure 5III,IV). CCI SHR rats (panel/column C-bis) spinal cord displayed more pronounced levels (*p* = 0.028) of 8-OHdG as compared to SHAM WKY and SHR rodents’ spinal cord (panel/column A and B, respectively), markedly in the cell body neurons of dorsal horns. Treatments with almost all forms of thioctic acid induced an antioxidant effect (panel/column D, E, F), only (−)-thioctic acid (panel/column G) injections produced no amelioration, similarly to pregabalin (panel/column H).

**FIGURE 6:**
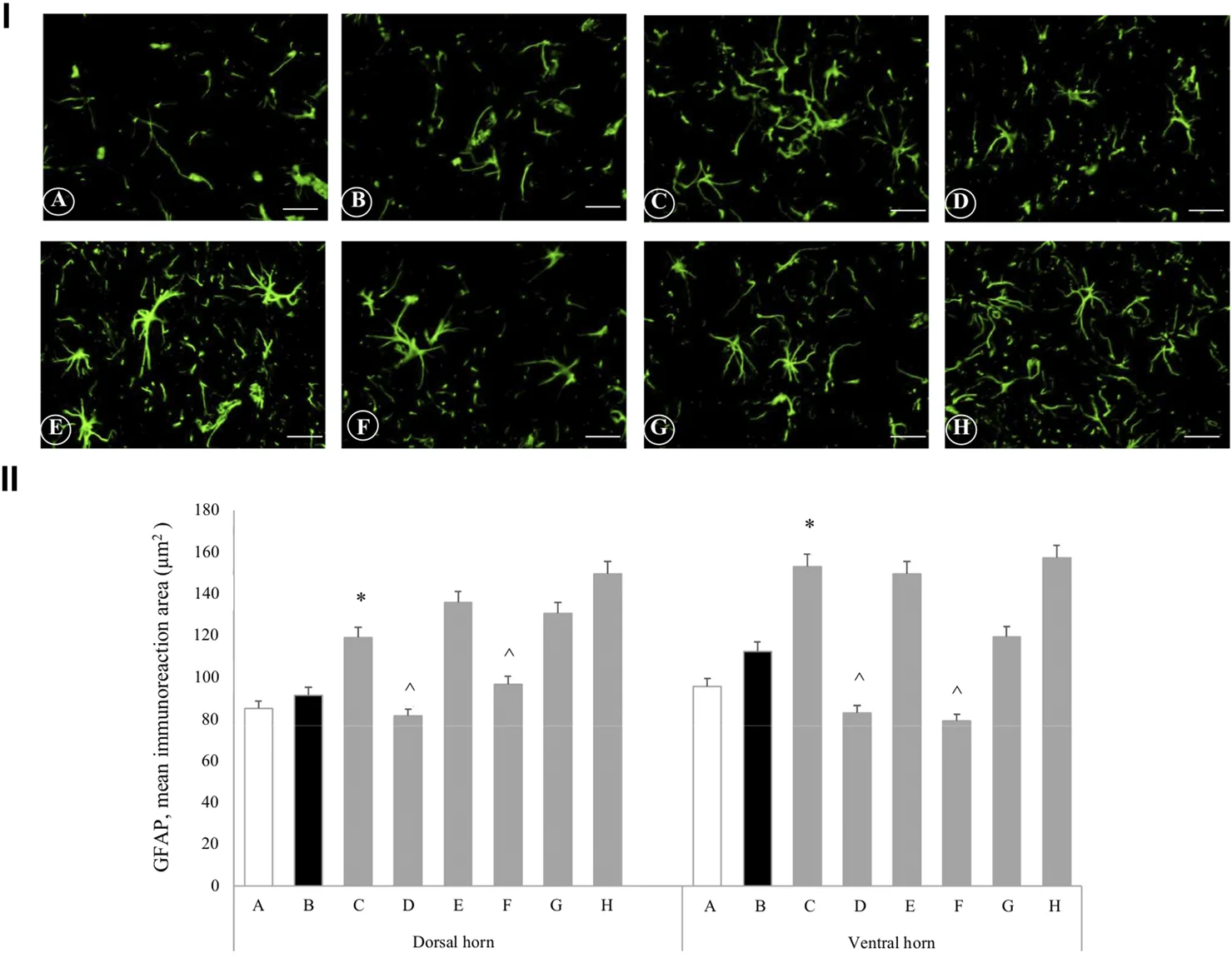
Astrocytes activation: GFAP immunohistochemistry in spinal cord. Sections of dorsal horn of spinal cord (images A-H, i) processed for the immunohistochemistry of glial fibrillary acid protein (GFAP) and densitometric analysis (ii) of the mean immunoreaction area in both dorsal and ventral horns. In all panels, treatments are indicated as follows: **(A)** SHAM WKY + saline solution; **(B)** SHAM SHR + saline solution; **(C)** CCI SHR + saline solution; **(D)** CCI SHR + (+/−)-thioctic acid 250 μmol·kg^−1^·die^−1^; **(E)** CCI SHR + (+/−)-thioctic acid 125 μmol·kg^−1^·die^−1^; **(F)** CCI SHR + (+)-thioctic acid 125 μmol·kg^−1^·die^−1^; **(G)** CCI SHR + (−)-thioctic acid 125 μmol·kg^−1^·die^−1^; **(H)** CCI SHR + Pregabalin 300 μmol·kg^−1^·die^−1^. Calibration bar: 25 μm. Data are expressed as mean ± SEM; *p < 0.05 vs SHAM SHR + saline solution; ^*p* < 0.05 vs CCI SHR + saline solution.

In addition, we evaluated the neuroprotective effects of thioctic acid from *ex-vivo* analysis on the spinal cord (Figures 6i–6ii). Dorsal horn sections were probed with GFAP antibody (Figure 6i): the number of GFAP-positive cells was comparable between SHAM SHR and WKY rats (panels A and B), therefore we moved on the analysis of SHR animals. Sciatic nerve ligation induced an activation of astrocytes with high production of GFAP and ramified branches (panel C), while treating with (+/−)-thioctic acid 250 μmol·kg^−1^·die^−1^ restored the physiological state (panel D). Injections with (+)-thioctic acid (panel F) reproduced the same result of double-concentrated racemic compound (panel D); conversely, an opposite effect was obtained by the treatment with 125 μmol·kg^−1^·die^−1^ racemic compound and (−)-enantiomer (panel E and G, respectively). A pattern of activated astrocytes was also observed in the spinal cord of pregabalin treated rats (panel H). These observations were confirmed by the analysis of mean area (μm^2^) of GFAP-immunopositive astrocytes, from both dorsal and ventral horns (Figure 6ii). No significant differences between the two regions were noticeable in each experimental group. In the dorsal horn, sham WKY and SHR rats presented the same quantity of immunoreactive tissue while CCI SHR animals had 30% more GFAP-positive tissue. Treatments with (+/−)-thioctic acid 250 μmol·kg^−1^·die^−1^ (*p* = 0.005 *vs.* CCI) and (+)-enantiomer 125 μmol·kg^−1^·die^−1^ (*p* = 0.032 *vs.* CCI) reduced the area almost to non-neuropathic values, while glial activation was confirmed in groups treated with 125 μmol·kg^−1^·die^−1^ racemic thioctic acid, (−)-enantiomer and pregabalin. In the ventral horn the pattern is the same.

### Clinical Results

#### Neuropathy Symptoms and Change (NSC) Parameter

Initial (baseline) values for the NSC scale averaged 17.2 ± 5.1 in Group 1 patients (+/−)-thioctic acid and 17.6 ± 5.3 in Group 2 patients (+)-thioctic acid. After 60 days of treatment, these values decreased to 13.0 ± 5.4 in group 1 patients and 11.3 ± 4.8 in group 2 patients (Table 2). This reduction was statistically significant vs. baseline for both treatments (respectively *p* = 0.0000 and *p* = 0.0000 for the two groups at the two-sided Student’s t test for paired data). The average reduction for this scale was of 4.12 ± 3.13 points for patients of Group 1 and 6.26 ± 4.30 points for patients of Group 2 (Table 2) (*p* = 0.0003, ANOVA). (Supplementary Figure S3A). Values of the NSC scale were also used to assess the percentage change compared to the starting value. Percentage changes resulted to be of 24.7 ± 17.3% in Group 1 patients and 34.4 ± 19.1% in Group 2 patients (Table 2) (*p* = 0.009, ANOVA). To analyze the different treatment over time a two way ANOVA for repeated measure was performed: data showed significative results for the model (*p* = 0.0000) and for the time (*p* = 0.0000) and treatment (*p* = 0.048) parameters.

**TABLE 2:** Neuropathy Symptoms and Change scale (NSC), Neuropathy Impairment Score (NIS) and Neuropathy Total Symptom Score-6 (NTSS-6) in Group 1 patients [(+/−)-thioctic acid)] and in Group 2 patients [(+)-thioctic acid] at baseline (T0) and after 60 days of treatment (Tlast).Data are means ± S.D. Response rate and pain half-life data represent the percentage of patients with reduction of pain score respect to baseline after treatment (response ratio) and the days for pain half-life.

Neuropathy symptoms and change scale (NSC), neuropathy impairment score (NIS) and neuropathy total symptom Score-6 (NTSS-6)			
Pain scales		Group 2 (+)-thioctic acid	Group 1 (+/−)-thioctic acid
NCS	T0	17.6 ± 5.3	17.2 ± 5.1
	Tlast	11.3 ± 4.8*^#^	13.0 ± 5.4*
Reduction from baseline		6.26 ± 4.30^#^	4.12 ± 3.13
Percentage reduction		34.4 ± 19.1^#^	24.7 ± 17.3
Response rate		94°	84
Pain half-life		84	125
NIS	T0	4.28 ± 4.22	4.30 ± 3.08
	Tlast	1.94 ± 3.80*	2.64 ± 2.99*
Reduction from baseline		2.34 ± 2.56	1.66 ± 1.81
Percentage reduction		53.9 ± 44.4	40.2 ± 43.3
Response rate		66	60
Pain half-life		55	78
NTSS-6	T0	3.86 ± 2.95	4.01 ± 2.56
	Tlast	1.98 ± 2.47*	2.65 ± 2.36*
Reduction from baseline		1.88 ± 1.96	1.35 ± 1.95
Percentage reduction		53.7 ± 40.2^#^	36.2 ± 39.7
Response rate		78°	64
Pain half-life		62	89

*p < 0.05 vs T0 evaluated by Student’s t-test for paired data; ^#^p < 0.05 vs Group 1 patients evaluated by ANOVA Test; ° p < 0.05 vs. Group 1 patients, X^2^ test.

#### Neuropathic Injury Score Parameter

Initial (baseline) average values for the NIS scale were 4.30 ± 3.08 in Group 1 patients and 4.28 ± 4.22 in Group 2 patients. After 60 days of therapy, values decreased to 2.64 ± 2.99 in Group 1 patients and 1.94 ± 3.80 in Group 2 patients (Table 2). This reduction was statistically significant *vs*. baseline for both treatments (*p* = 0.0000 for both groups at two sided Student's t test for paired data). The average reduction in the scale was of 1.66 ± 1.81 points in Group 1 patients and 2.34 ± 2.56 points for Group 2 patients (Table 2). This reduction did not reach statistical significance at the ANOVA (*p* = 0.308). (Supplementary Figure S3B). Values of NIS were also evaluated in terms of percent reduction compared to the starting value: the decrease, which averaged 40.2 ± 43.3% in Group 1 patients and 53.9 ± 44.4% in Group 2 patients (Table 2), was not statistically significant (*p* = 0.121). Results of the two way ANOVA for repeated measure showed significative results for the model (*p* = 0.0000) and for the time (*p* = 0.0000) but not significative for treatment (*p* = 0.474) parameter.

#### Neuropathy Total Symptom Score-6 (NTSS-6) Parameter

Initial (baseline) average values for the NTSS-6 scale were 4.01 ± 2.56 in Group 1 patients and 3.86 ± 2.95 in Group 2 patients. After 60 days of treatment these values decreased to 2.65 ± 2.36 in Group 1 patients and 1.98 ± 2.47 in Group 2 patients (Table 2). This reduction was statistically significant vs. baseline for both treatments (*p* = 0.0000 for both groups at two sided Student’s t test for paired data). The average reduction in the scale was of 1.35 ± 1.95 points for Group 1 patients and 1.88 ± 1.96 points for Group 2 patients (Table 2). This decrease was not significant at the ANOVA analysis (*p* = 0.165). (Supplementary Figure S3C). Data were also evaluated as percent reduction compared to the baseline: this analysis resulted in the 36.2 ± 39.7% in the Group 1 patients and in the 53.7 ± 40.2% in the Group 2 patients (Table 2) (*p* = 0.031, ANOVA). Results of the two way ANOVA for repeated measure showed significative results for the model (*p* = 0.0000) and for the time (*p* = 0.0000) but not significative for treatment (*p* = 0.264) parameter.

#### Quality of Life

Both treatment with thioctic acid were safe, no patients withdrawn from study; of the 107 patients considered, 7 did not meet the inclusion criteria (first manifestation, presentation before 40 days). Of the patients only one experienced side effects after about a month of treatment, with loss of taste and a sense of bitterness in the mouth. The manifestation was considered a “possible” side effect, but did not lead to discontinuation of therapy and ended before the last visit of the study. Considered quality of life criteria included the response rate to therapy, the half-lives of pain, changes in sleep quality and changes in painkillers assumption. As to NSC parameter, the response rate to therapy was 84% in Group 1 patients (+/−)-thioctic acid and 94% in Group 2 patients (+)-thioctic acid. As to NIS parameter, the response was positive in 60% of patients of Group 1 and in 66% patients of Group 2. For NTSS-6 scale, a positive response to therapy was observed in 64% patients of Group 1 and in 78% patients of Group 2 (Table 3).

**TABLE 3:** Sleep quality and pain killer intake variations in Group 1 [(+/−)-thioctic acid)] and in Group 2 [(+)-thioctic acid]. Data shown represent the absolute number and the percentage of patients.

**Sleep quality and pain killer intake variations**			
**Quality of life**	**Group 2**	**Group 1**
	**(+)-thioctic acid**	**(+/−)-thioctic acid**	**Significance at the X squared test**
**Quality of sleep**		
Improved	33 (66%)	24 (48%)	*p* = 0,00,097
Unchanged	17 (34%)	25 (50%)	
Worsened	0 (0%)	1 (2%)	
Painkiller intake			
Increased	1 (2%)	1 (2%)	*p* = 0,01,593
Unchanged	13 (26%)	20 (40%)	
Reduced	36 (72%)	29 (58%)	

Half-life of pain, representing the time during which the parameters were considered, reached half of the initial value: for NSC it was 125 days in Group 1 patients and 84 days in Group 2 patients with a 32% reduction for (+)-thioctic acid as compared to (+/−)-thioctic acid. The half-life parameter for NIS was 78 days in Group 1 patients and 55 days in Group 2 patients, with a 29% reduction for (+)-thioctic acid as compared to (+/−)-thioctic acid. Assessment of half-lives for the scale NTSS-6 resulted in 86 days in Group 1 patients and 62 days in Group 2 patients, with a reduction of 29% in favor of (+)-thioctic acid (Table 3).

Assessment of sleep quality showed in Group 1 patients a 48% of sleep improvement, a 50% of lack of difference and a 2% of worsening. In Group 2 patients, a 66% of sleep improvement, a 34% of no differences and no cases of worsening were reported (Table 3).

Analysis of the need to add painkiller to the antioxidant-based therapy revealed in Group 1 patients a reduction of analgesics intake in 58% of individuals, no changes in 40% of them and an increase in 2% of subjects. In Group 2, 72% of patients reduced the use of painkillers, which remained unchanged in 26% of cases. In 2% of examined patients, analgesic medication was increased (Table 3).

#### Gender Influence Evaluation

The stratification of data by gender showed that the groups were mostly homogeneous in the distribution with regard to both sex and age (Group 1: 24 female, mean age 53.0 ± 10.0; 26 male, mean age 53.5 ± 12.5; Group 2: 27 female, mean age 54.6 ± 11.4; 23 male, mean age 52.7 ± 11.4)

The analysis performed by two-way ANOVA for repeated data considering the variables of sex and treatment showed at the end of the study that none of the three pain parameters considered were influenced by the sex of the participants. (NSC: overall value *p* = 0.006, treatment *p* = 0.005, gender *p* = 0.172; NIS: overall value *p* = 0.403, treatment *p* = 0.335, gender *p* = 0.377; NTSS-6: overall value *p* = 0.379, treatment *p* = 0.171, gender *p* = 0.876).

## Discussion

Low back pain is becoming one of the most common diseases in industrialized countries, due to inappropriate postural attitudes and sedentary lifestyles: more than 70% of individuals are estimated to suffer of low back pain at least one time in their life (Baron et al., 2016); it is the fifth reason for medical consultation in the United States (U.S.) and about a quarter of U.S. adults experienced low back pain for at least one whole day over a period of three months (Deyo et al., 2006). The guidelines of the European Federation of Neurological Societies (EFNS) and of the International Association for the Study of Pain (IASP) have been considering the use of different classes of drugs, such as analgesics, antidepressants and anticonvulsants, for the treatment of neuropathic pain (Dworkin et al., 2007; Attal et al., 2010).

A proper treatment of low back pain should not only control pain, but also maintain/restore nerve function. Oxidative stress reduces neuronal function and local blood flow, limiting the arrival of nutrients to nerve cells (Mitsui et al., 1999; Memeo and Loiero, 2008). Antioxidant products, therefore, could contribute to control symptoms and act on the pathogenesis as well (Ranieri et al., 2009). Thioctic acid is a natural fatty acid endogenously produced by mammalian cells and chemically existing as two optical isomers. It is an essential component of some mitochondrial enzyme complexes, important in the glucose metabolism (de Arriba et al., 2003) and able to actively counter various forms of oxidative stress (Tibullo et al., 2017). The dextrorotatory enantiomer is better recognized by enzymes (Streeper et al., 1997) and maximum plasma concentration (Cmax) is approximately 40–50% higher with (+)-thioctic acid than with (+/−)-thioctic acid at the same dose (Carlson et al., 2007).

Preclinical experiments on CCI-SHR showed that following a 15-days cure with thioctic acid (racemic form, dextrorotary and levorotary enantiomers), neuropathic hypertensive rats ameliorated their altered algesic sensitivity and oxidative stress levels. Moreover, the treatment with (+)-enantiomer was as effective as with double-concentrated racemic (+/−)-thioctic acid and the nociceptive threshold closely reached that obtained by administrating pregabalin; on the contrary, the levorotatory enantiomer alone was as ineffective as injecting the saline solution. All these data suggested a prominently active role of the dextrorotatory enantiomer in the racemic mixture. Accordingly, previous studies on chemotherapy-induced neuropathy described similar results: thioctic acid acutely dosed to vincristine-treated rats reversed allodynia symptoms; chronic injections of 15, 30, and 60 mg/kg of (+/−)-thioctic acid on neuropathic rodents, which were given paclitaxel, significantly reduced mechanical and cold allodynia (Kahng et al., 2015; Sun et al., 2019). As previously reported (Tomassoni et al., 2013), SHR rats showed higher values of systolic blood pressure as compared to WKY rats. CCI did not induce an increase of blood pressure values, nor different formulations of racemic and enantiomer thioctic acid affected blood pressure values in SHR. These results demonstrated that the analgesic and neuroprotective effects of thioctic acid was not mediated by a decrease of systolic blood pressure (Tomassoni et al., 2013).

Microanatomical analysis of this study revealed changes after loose ligation of the sciatic nerve: either axonal components of the nerve and myelin sheaths were affected, involving myelinated and unmyelinated nerve fibers. These findings support and extend previous studies reporting the degeneration of axonal components and myelin sheaths, with decrease of NF and MBP in the portion of nerve which is distal to the ligation (Di Cesare Mannelli et al., 2009; Tomassoni et al., 2018).

Thioctic acid, above all the (+)-enantiomer form, exerted a protective activity on the peripheral nerve portion affected by ligation, not shared by pregabalin. We would suppose that amelioration of hyperalgesia after treatment may partly depend on the effects that thioctic acid induced on sciatic nerve morphology, due to the antioxidant ability to scavenge and inactivate free radicals. Its supplementation as natural antioxidant has already demonstrated multiple beneficial effects (Tibullo et al., 2017) and (+)-thioctic acid showed the most pronounced activity.

Studies about the mechanisms of neuropathic pain following injury of peripheral nerves demonstrated that nerve damage was related to altered neuronal plasticity and aberrant function of glial cells in the lumbar spinal cord (Cirillo et al., 2015; De Luca et al., 2016). These neuroglial plastic changes induce both neuronal/astrocytic activation and alteration of neuroglial interactions, determining maladaptive synaptic plasticity in the spinal somatosensory system, which seems to be directly responsible for the neuronal hyperexcitability and the enhanced synaptic transmission that sustain neuropathic pain (Gwak and Hulsebosch, 2009; Wang et al., 2009).

As previously demonstrated (Cirillo et al., 2011, Cirillo et al., 2012; Colangelo et al., 2012), our evidence confirms that in the lumbar spinal cord CCI is associated with reactive gliosis, characterized by hypertrophy of astrocytes and their activation, and with an increase of oxidative stress phenomena in the somatosensory neurons of the dorsal horn. Here, the beneficial effects of thioctic acid were related to its antioxidant properties, like the ability to restore the intrinsic antioxidant systems, supporting their production or cell accessibility (Shay et al., 2009; Goraca et al., 2011; Salehi et al., 2019). The compound, in particular its dextrorotatory enantiomer, lowered the oxidation status of proteins and the expression of 8-OHdG in the somatosensory neurons of the dorsal horn: it might be, in a positive loop, linked to effects on astrocytes cells, since treatments with dextrorotatory enantiomer and double-concentrated racemic formulation reduced size of astrocytes and GFAP expression.

These data from spinal cord could explain the protection of (+)-thioctic acid on the brain. In a previous study we indicated, after mono-lateral CCI of sciatic nerve, augmented GFAP expression mainly in the gray matter of sensory cortex and decreased NF expression as a consequence of nerve damage; we also showed that treatment with antioxidants, but not with pregabalin, prevented to some extent astrogliosis and neuronal damage in cerebral cortex, similarly to the present findings in spinal cord (Tomassoni et al., 2013).

Encouraged by the findings of the preclinical study, which pointed out a more prominent activity of (+)-thioctic acid as compared to the other tested forms of thioctic acid, resembling or even exceeding the efficacy of pregabalin, a clinical study was set up (using a dosage comparable to the preclinical evaluation, Reagan-Shaw et al. (2008) and Nair and Jacob (2016). The results of the clinical study confirmed the efficacy of the antioxidant thioctic acid in the treatment of the peripheral neuropathy without differences in male and female (Memeo and Loiero, 2008; Ranieri et al., 2009; Agathos et al., 2018; Mrakic-Sposta et al., 2018; Salehi et al., 2019; Passiatore et al., 2020) being also characterized by good safety profile which makes it suitable for prolonged treatment even in the chronic phase of this disease (Ametov et al., 2003; Ziegler et al., 2006). Anyway, it should be carefully prescribed and monitored since the possibility of side effects as recently emerged in a preclinical toxicologic study (Lucarini et al., 2020). The results of clinical study show a greater effectiveness of (+)-thioctic acid compared to (+/−)-thioctic acid in terms of major impact on pain symptoms, rapidity of therapeutic effects onset and, more generally, better quality of life, as confirmed by the response rate to therapy. Our results are consistent with the effects observed in the same period of time (60 days) in other studies (Mrakic-Sposta et al., 2018; Passiatore et al., 2020), even if with minor side effects with respect to those observed in patients treated with a dose higher than 600 mg/day (Mrakic-Sposta et al., 2018). The advantage in using (+)-thioctic acid, as compared to the racemic form, may be related to an increased bioavailability of this enantiomer, that boosts its antioxidant activity (Maglione et al., 2015) as well as to higher biological activity. Preclinical and clinical evidences suggest positive properties of thioctic acid in the treatment of low back pain with a more relevant efficacy of (+)-thioctic acid compared to (+/−)-thioctic acid on pain, on time of onset of therapeutic effects and on quality of life of patients suffering from the symptoms under study. These observations are worthy of further analysis, but they make it a good candidate for treatment of low back pain.

## Data Availability Statement

The raw data supporting the conclusion of this article will be made available by the authors, without undue reservation.
