# Correction: Comparative assessment of the activity of racemic and dextrorotatory forms of thioctic (alpha-lipoic) acid in low back pain: preclinical results and clinical evidences from an open randomized trial

**DOI:** 10.3389/fphar.2026.1896081

**Published:** 2026-09-02

**Authors:** Alessandra Pacini, Daniele Tomassoni, Elena Trallori, Laura Micheli, Francesco Amenta, Carla Ghelardini, Lorenzo Di Cesare Mannelli, Enea Traini

**Affiliations:** 1 Department of Experimental and Clinical Medicine, Anatomy and Histology Section, University of Florence, Florence, Italy; 2 School of Biosciences and Veterinary Medicine, University of Camerino, Camerino, Italy; 3 Department of Neuroscience, Psychology, Drug Research and Child Health (NEUROFARBA)-Pharmacology and Toxicology Section, University of Florence, Florence, Italy; 4 Section of Human Anatomy, School of Pharmacy, University of Camerino, Camerino, Italy

**Keywords:** neuropathic pain, thioctic acid, antioxidant, food supplement, neuroprotection

There was a mistake in Figure 5I, as published. Inadvertently, during figure preparation, an incorrect image was used as the representative Western blot. The corrected Figure 5 appears below.

There was a mistake in the caption of Figure 5 as published. The word “carbonylated” was misspelled, and panels were indicated with i), ii), iii), iv) instead of I), II), III), IV) as in the figure. The corrected caption of Figure 5 appears below.

The original version of this article has been updated.

**FIGURE 5:**
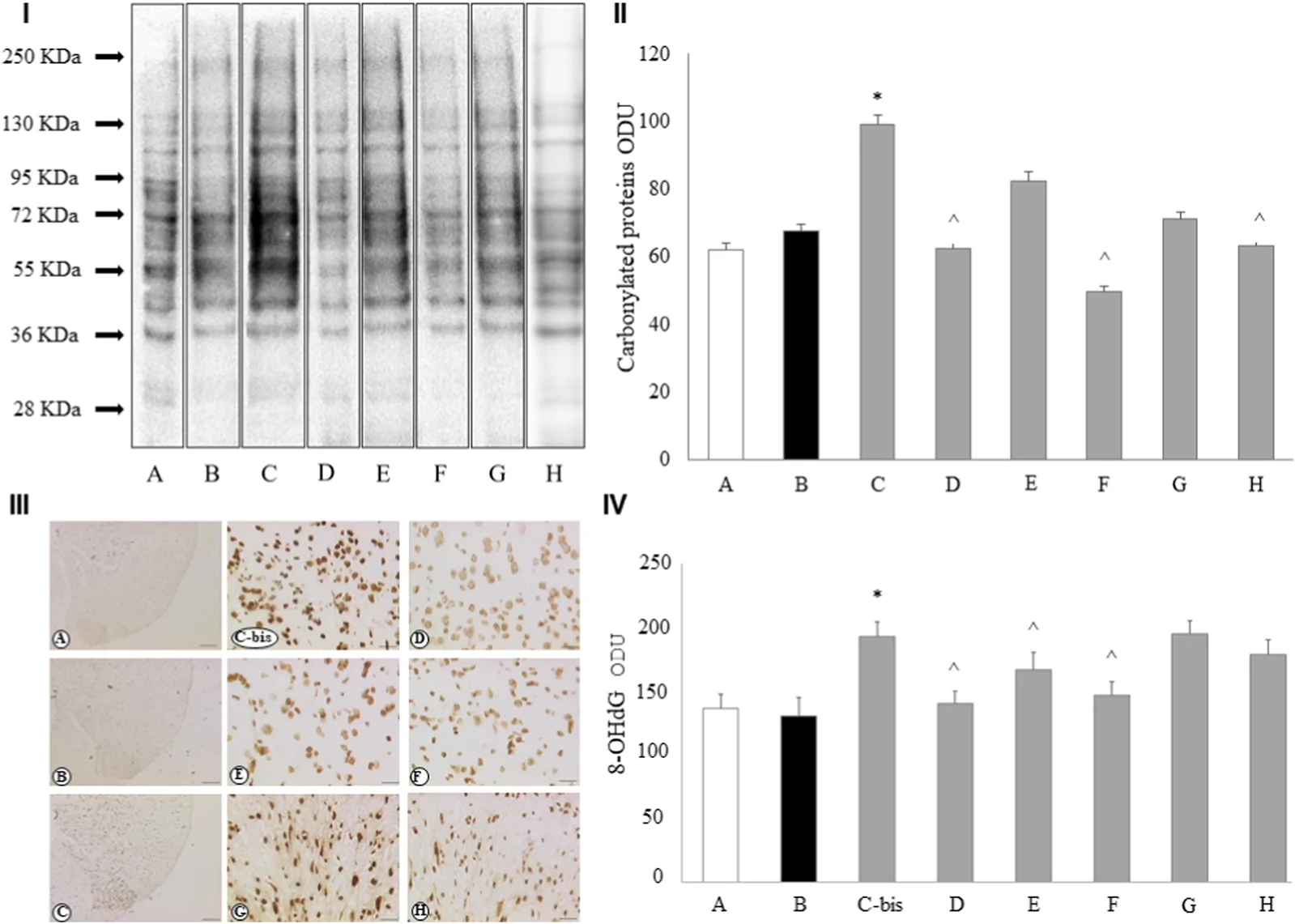
Oxidative stress status in the spinal cord (lumbar region L5). Carbonylated proteins blotting of the spinal cord **(I)** and its densitometric analysis **(II)**. Sections of dorsal horns of spinal cord (lumbar region L5) processed for immunohistochemistry of 8-OHdG **(III)** and densitometric analysis **(IV)**. Calibration bar of **(III)**: A-C 200 μm; Cbis-H: 25 μm; densitometric analysis was performed on 25 μm calibration bar-images. In all panels, treatments are indicated as follows: (A) SHAM WKY + saline solution; (B) SHAM SHR + saline solution; (C) Cbis) CCI SHR + saline solution; (D) CCI SHR + (+/−)-thioctic acid 250 µmol⋅kg–1⋅die^−1^; (E) CCI SHR + (+/−)-thioctic acid 125 μmol⋅kg^−1^⋅die^−1^; (F) CCI SHR + (+)-thioctic acid 125 μmol⋅kg^−1^⋅die^−1^; (G) CCI SHR + (−)-thioctic acid 125 μmol⋅kg^−1^⋅die^−1^; (H) CCI SHR + Pregabalin 300 μmol⋅kg^−1^⋅die^−1^. Data are expressed as mean ± SEM. *p < 0.05 vs. SHAM SHR + saline solution; ^*p* < 0.05 vs. CCI SHR + saline solution.

