# Supplementary material for: Comparative Assessment of the Activity of Racemic and Dextrorotatory Forms of Thioctic (Alpha-Lipoic) Acid in Low Back Pain: Preclinical Results and Clinical Evidences From an Open Randomized Trial
Source: Front Pharmacol. 2021 Feb 24;12:607572. doi: 10.3389/fphar.2021.607572 (PMC7959756; doi:10.3389/fphar.2021.607572)
Supplement: Supplementary file 1 [file DataSheet1.docx]

Supplementary Material

# 1 Supplementary Figures

**Supplementary Figure** **1****.**Mallory’s trichrome staining of sciatic nerve. Section of sciatic nerve processed for Mallory’s trichrome staining. A) CCI SHR + saline solution; B) CCI SHR + (+/-)-thioctic acid 125 μmol‧kg^-1^‧die^-1^; C) CCI SHR + (+/-)-thioctic acid 250 μmol‧kg^-1^‧die^-1^; D) CCI SHR + (+)-thioctic acid 125 μmol‧kg^-1^‧die^-1^; E) CCI SHR + (-)-thioctic acid 125 μmol‧kg^-1^‧die^-1^; F) CCI SHR + Pregabalin 300 μmol‧kg^-1^‧die^-1^. Calibration bar: 10 μm. 

**Supplementary Figure 2.** Malondialdehyde (MDA) levels in spinal cord. Levels of Malondialdehyde in the spinal cord omogenates, evaluated by TBARS kit. A) SHAM WKY + saline solution; B) SHAM SHR + saline solution; C) CCI SHR + saline solution; D) CCISHR +(+/-)-thioctic acid 250 μmol‧kg^-1^‧die^-1^; E) CCI SHR + (+/-)-thioctic acid 125 μmol‧kg^-1^‧die^-1^; F) CCI SHR + (+)-thioctic acid 125 μmol‧kg^-1^‧die^-1^; G) CCI SHR + (-)-thioctic acid 125 μmol‧kg^-1^‧die^-1^; H) CCI SHR + Pregabalin 300 μmol‧kg^-1^‧die^-1^. Data, express as pmol/mg of tissue, are the mean ± SEM. *p<0.05 vs SHAM SHR + saline solution.

**Supplementary Figure 3.** Scales NSC (A), NIS (B) e TSS (C) scores in Group 1 patients [(+/-)-thioctic acid)] and in Group  2 patients [(+)-thioctic acid] at baseline (T0) and after 60 days of treatment (Tlast). Data are means ± S.E.M.  *p<0,05 vs T0 evaluated by Student’s t-test for paired data; #p<0,05 vs Group 1 patients evaluated by ANOVA Test.
